# Cost-utility analysis of trabecular micro-bypass stents (TBS) in patients with mild-to-moderate open-angle Glaucoma in Italy

**DOI:** 10.1186/s12913-021-06862-x

**Published:** 2021-08-17

**Authors:** Antonio Maria Fea, Francesco Cattel, Stefano Gandolfi, Giorgio Buseghin, Gianluca Furneri, Ciro Costagliola

**Affiliations:** 1 Department of Surgical Sciences, University of Turin, Turin, Italy; 2 Department of Medical Sciences, University of Turin, Amedeo di Savoia Hospital, City of Science and Health Molinette, Hospital Pharmacy, Turin, Italy; 3 Ophthalmology Unit, Department of Biological, Biotechnological and Translational Sciences, University of Parma, Parma, Italy; 4 Glaukos, San Clemente, California USA; 5 EBMA Consulting SRL, Melegnano, Milan, Italy; 6 Department of Medicine & Health Sciences “V. Tiberio”, University of Molise, Campobasso, Italy

**Keywords:** Cost-effectiveness, Minimally invasive surgery, glaucoma, IOP reduction, Trabecular bypass

## Abstract

**Background:**

Glaucoma is a disease characterized by progressive damage of the optic nerve. Several therapeutic options are available to lower intraocular pressure (IOP). In primary open-angle glaucoma (POAG) patients with inadequate IOP control (or controlled with multiple medical therapies or for whom medical therapy is contraindicated), the implantation of micro-invasive glaucoma surgery devices (MIGS) and concomitant cataract surgery has proved to be more effective in reducing intraocular pressure (IOP), as compared to cataract surgery alone. The objective of this study was to assess the cost-effectiveness of iStent *inject*® device with concurrent cataract surgery vs. cataract surgery alone, in patients with mild-to-moderate POAG, adopting the Italian National Health Service (NHS) perspective.

**Methods:**

Simulation of outcomes and costs was undertaken using a Markov model with 4 health states and one-month cycles, that is used to simulate the prognosis of these patients. Efficacy data were obtained from the randomized clinical trial (RCT). A lifetime horizon was adopted in the analysis. A discount rate of 3.5% was applied to both costs and effects. The Italian National Healthcare Service (NHS) perspective was considered, therefore only healthcare direct costs (acquisition of main interventions and subsequent procedures; medications; monitoring and follow-up; adverse events). Model robustness was tested through sensitivity analyses.

**Results:**

Results of the base-case analysis showed that the total lifetime costs were higher in the iStent inject® + concurrent cataract surgery, compared with the cataract surgery alone group (€8368.51 vs. €7134.71 respectively). iStent inject® + concurrent cataract surgery was cost-effective vs. cataract surgery alone, with an incremental cost-effectiveness ratio of €13,037.01 per quality-adjusted life year (QALY) gained. Both one-way deterministic and probabilistic sensitivity analyses confirmed robustness of base-case results. The cost-effectiveness accessibility curve (CEAC) showed that iStent inject® + cataract surgery would have a 98% probability of being cost-effective, compared to cataract surgery alone, when the willingness to pay (WTP) is equal to €50,000 per QALY gained.

**Conclusions:**

The results of the cost-utility analysis confirm that iStent inject® + cataract surgery is a cost-effective option for the treatment of patients affected by mild-to-moderate POAG, compared with cataract surgery alone, when evaluated from the Italian NHS perspective.

**Supplementary Information:**

The online version contains supplementary material available at 10.1186/s12913-021-06862-x.

## Background

Glaucoma is a disease characterized by progressive damage of the optic nerve [1] and is the second cause of blindness globally, after cataract [1]: it affects approximately 66.8 million people worldwide [2, 3]. Recent estimates show that about 1 million subjects suffering from glaucoma in Italy; only half of them have a confirmed diagnosis [1]. Every year, about 4500 new cases of blindness are registered in Italy, and approximately 200,000 blindness cases in total are correlated to this pathology [4].

Primary open-angle glaucoma (POAG) is the most common form of glaucoma, accounting for about 90% of all glaucoma cases [5]. Patients with mild-to-moderate POAG may have significant visual disability, with impairment of their visual field, contrast sensitivity, and light-to-dark and dark-to-light adaptation.

Intraocular pressure (IOP) is the only modifiable risk factor for glaucoma [6]. An abnormality in the trabecular meshwork is the primary cause of reduced aqueous outflow, and therefore of increased IOP. Several therapeutic options are available to lower IOP, including medical therapy, laser trabeculoplasty (ALT/SLT), non-filtering micro-invasive glaucoma surgery (MIGS), anterior filtering surgeries, posterior filtering surgeries [7]. The choice of the optimal therapeutic intervention generally depends on several factors: the IOP level to be targeted, severity of glaucomatous damage induced by POAG, the disease progression rate, age of the patient, presence of comorbidities and level of ocular inflammation. Cataract and glaucoma frequently occur together, and their concomitant presence increases with age [8, 9]. the The American Glaucoma Society estimated that cataract surgery alone is the preferred initial surgical approach for 44% ± 32% of patients with POAG and visually significant cataract [10].

In patients with medically controlled, non-severe glaucoma and cataract, small-incision cataract extraction (phacoemulsification) alone may be a valid option to reduce IOP and to control glaucoma progression [11, 12]. However, in many patients with POAG (inadequate IOP control, multiple medications or intolerance to medical therapy), phacoemulsification alone could be insufficient to control POAG progression adequately.

In these subjects, the implantation of MIGS devices and concomitant cataract surgery has been shown to be more effective in reducing intraocular pressure (IOP), as compared to cataract surgery alone [13]. The IOP lowering effect after cataract surgery may be short- lived compared to when MIGS and cataract surgeries are combined [5, 14, 15]. Nowadays several MIGS are marketed including Sclemm’s canal stents (iStent®, iStent *inject*®, iStent *inject*® W, Hydrus®) and subconjunctival stents (Xen®, Innfocus®).

Among MIGS, the trabecular bypass stent (TBS) seems to have an optimal risk-benefit profile in patients with mild-to-moderate POAG.

The iStent *inject*® (Glaukos Corp., San Clemente, CA) TBS device is inserted into the trabecular meshwork through a single corneal incision to improve physiologic aqueous outflow. iStent *inject*® has been shown to lower IOP while reducing ocular hypotensive medication usage in prior studies comparing eyes randomly assigned to cataract surgery and iStent implantation or cataract surgery alone with follow-up through 12 months, up to 48 months [16–19]. While many studies have demonstrated the efficacy of MIGS, there is limited knowledge about the economic implications of MIGS implantation, in patients with POAG, in need of cataract surgery [20, 21]. The objective of this study is to assess the cost-effectiveness of iStent *inject*® device with concurrent cataract surgery vs. cataract surgery alone, in patients with mild-to-moderate POAG, adopting the Italian National Health Service (NHS) perspective.

## Methods

### Model design

**Fig. 1:**
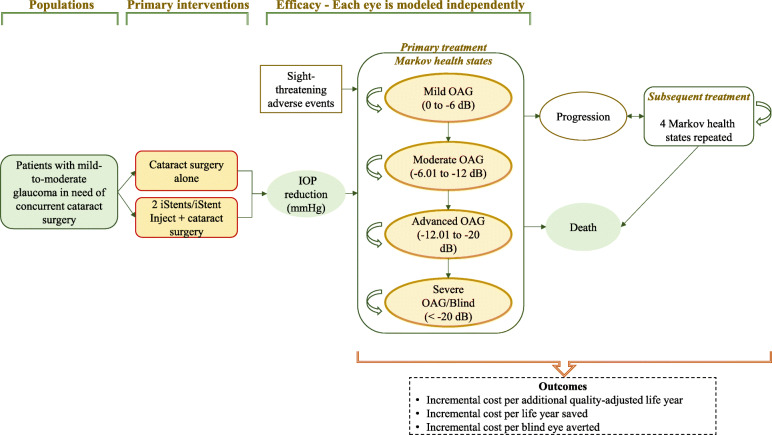
Scheme of the Markov model. *dB = decibel; IOP = intraocular pressure; OAG = open-angle glaucoma*

Our cost-effective analysis model compared the costs and clinical outcomes in patients undergoing cataract surgery and iStent *inject®* vs cataract surgery alone (which is the current standard of care in Italy) in one eye of patients with POAG over their lifetime. A previously published Markov model [22, 23], with 4 health states (Fig. 1) and one-month cycle, was adapted to simulate the prognosis of these patients. The model estimated the clinical benefits (in terms of quality-adjusted life years, QALY) and costs associated with the two different alternatives.

The model is based on the assumption that both quality of life outcomes and disease management costs of a hypothetical POAG patient would depend on the severity of the disease, defined according to the visual field (VF) based Hodapp-Parrish-Anderson scale [24]: i) mild: from 0 to − 6 dB; ii) moderate: from − 6.01 to − 12 dB; iii) advanced: − 12.01 to − 20 dB; iv) severe / blindness:> − 20 dB.

Each patient was allocated to one of the four glaucoma stages shown in Fig. 1. The disease progression rate (through more severe health states) was based on the natural history of glaucoma progression (dB) if left untreated. In real world, progression rates decline with treatment, which can reduce IOP. Transitions towards more severe health states determine an increase of resource consumption (more frequent consultations and tests) and a progressive reduction of patients’ utilities. The VF deterioration associated with the progression of glaucoma is irreversible; in the model, health state transitions would occur only to states of greater severity. Due to the relatively short duration of the cycles, it was assumed that it is not possible for patients to jump (skip) a health state (e.g. from mild to advanced or from moderate to severe health state).

In the model, patients with deteriorating VF would require trabeculectomy would require subsequent treatment, which is trabeculectomy. The risk of switching to trabeculectomy is expressed as a time-dependant function, whose shape depends on the IOP-modifying effect of first-line treatment (basically, the larger the IOP reduction the lower the proportion of patients who would need trabeculectomy).

Furthermore, patients may die at any time, from any of the other health states.

The model was developed in Microsoft Excel.

A lifetime horizon was adopted in the analysis. A discount rate of 3.5% was applied to both costs and effects. The Italian NHS (National Healthcare Service) payer perspective was adopted, i.e. considering only direct healthcare resources reimbursed and funded by the NHS.

### Clinical inputs

#### Characteristics of patients at baseline

At model start, patient population (POAG patients in need of cataract surgery) was aged 64.7 years (SD 12.1) as in the Traverso et al. 2005 clinical trial [25]. Unlike age, gender and ethnicity distributions were not considered, as it was supposed these would not influence clinical outcomes. It was our assumption that patients receiving iStent *inject*® had mild or moderate glaucoma (50.0% of patients with mild POAG, 50.0% of patients with moderate POAG) [26]. The complete list of parameters included in the analysis is available in Additional file 1.

#### Treatment effectiveness

In the model, effectiveness of the two treatment arms depends on two factors: i) IOP reduction obtained with surgical treatment (iStent inject® + cataract surgery vs. cataract surgery alone); ii) IOP reduction obtained with concomitant medical treatment.

Table 1 shows IOP levels and IOP reduction over time for the two alternatives. IOP data at one and 2 years after surgery were obtained from the randomized clinical trial (RCT) conducted by Samuelson et al. 2019 [16]. Samuelson et al. 2019 [16] is the first randomized trial comparing this second-generation iStent inject® device along with cataract surgery vs. surgery alone in POAG patients. Since efficacy of treatments is expected to decrease overtime, a 6.7% reduction of clinical effectiveness per year was hypothesized in both treatment arms (based on the estimates provided by the panel of experts) [26], to evaluate the efficacy of the interventions over the time horizon.

**Table 1:** Efficacy inputs with medication discontinuation at 8.6 months [Source: elaborated from Samuelson et al 2019 [16]]

Time (years)	iStent + Cataract surgery		Cataract surgery	
	IOP (mmHg)	IOP reduction (mmHg)^**a**^	IOP (mmHg)	IOP reduction (mmHg)^**a**^
0	24.8^b^	–	24.5**	–
1	17.7	7.1^b^	19.0	5.5^b^
2	18.3	6.6^b^	20.1	4.4^b^
3	19.1	5.7	20.9	3.6
4	19.4	5.4	21.2	3.3
5	19.7	5.1	21.3	3.2
6	20.0	4.8	21.5	3.0
7	20.3	4.5	21.7	2.8
8	20.5	4.3	21.8	2.7
9	20.8	4.0	22.0	2.5
10	21.0	3.8	22.1	2.4
10+	21.2	3.6	22.2	2.3

*IOP* intraocular pressur

Values after 2 years are based on expert opinions

^a^Intraocular pressure reduction vs. baseline

^b^IOP and IOP reduction data, at one and two years, were obtained from the randomized clinical trial (RCT) conducted by Samuelson et al 2019 [16] and adjusted for the time-to-discontinuation. Baseline IOP: 24.8 ± 3.3 mmHg vs. 24.5 ± 3.1 mmHg in the iStent + Cataract surgery vs. Cataract surgery groups, respectively (*P* = 0.33)

Beyond surgical treatment, IOP can be also reduced with concomitant medical therapy. Treatment effectiveness depends on the number of medications used. The progressive reduction of medication use was modelled, for the two alternatives, by incorporating the discontinuation rate of medical therapy [27]. The weighted average time-to-discontinuation was 8.6 months, according to Nordstrom et al. 2005 [28]. It was also assumed that the IOP increase due to discontinuation was equal to the difference in baseline medicated versus unmedicated IOP (7 mmHg), derived from Samuelson et al. 2011 [29].

As mentioned earlier, IOP reduction has the effect of reducing the risk of VF decline, measured with the Hodapp-Parrish-Anderson scale. If glaucoma were untreated, VF monthly decline would be -0.0508 dB [30]. Instead, if treated, one-unit reduction in IOP (mmHg) would determine a 9.5% decrease of the VF decline, according to the Early Manifest Glaucoma Trial (EMGT) [30]. These assumptions were used to determine transition probabilities between mild and moderate, moderate and advanced, advanced and severe health states will depend on IOP reduction.

Furthermore, IOP reduction has the effect of reducing the risk of VF disease progression, which is used in the model to estimate the proportion of patients who would require trabeculectomy. The natural disease progression in glaucoma patients has been described by Heijl et al. [30] and can be assimilated to a lognormal distribution. From published literature it was observed that one-unit reduction in IOP (expressed in mmHg) was associated with a 12% reduction of the risk of disease progression, compared with the natural history of the disease (hazard ratio: 0.88 [31]).

#### Efficacy of subsequent treatments

In the model, it was assumed that patients experiencing disease progression received subsequent treatment. According to Italian expert opinion, the most plausible treatment following cataract surgery (with or without MIGS implantation) was trabeculectomy. Efficacy data for trabeculectomy, expressed in terms of IOP reduction, were obtained from an indirect comparison analysis conducted by the National Institute of Health Care and Excellence (NICE) [32]. According to this source, trabeculectomy reduced IOP of 6.48 mmHg.

#### Mortality

At any time and health state, patients could move to the death health state. However, it was assumed that glaucoma would not modify (i.e. increase) the risk of death. For this reason, mortality probabilities were obtained from the general mortality tables of the Italian population (source: Italian Institute of Statistics, ISTAT; year 2017 [33]).

### Cost inputs

To provide a thorough assessment of the economic impact of glaucoma management, the following costs were included in the analysis: i) costs associated with main interventions (glaucoma + cataract surgery and cataract alone surgery); ii) costs of subsequent procedures; iii) costs of medications; iv) costs of monitoring and follow-up; v) costs of adverse events.

Table 2 lists all cost input data and resource consumption assumptions used in the model.

**Table 2:** Cost input included in the analysis

Type	Description	Value	Source
Main procedures	Glaucoma + cataract surgery cost (€)	€2294.20	iStent acquisition cost + procedures cost [34–37]
	Cataract surgery cost (€)	€994.00	DH 39 [38]
Subsequent procedures	Trabeculectomy cost (€)	€1969.10	Code 12.64 [35, 39, 40]
Medications	Bimatoprost cost (€)	€20.23	Generic ex-factory price [41]
	Bimatoprost + timolol cost (€)	€27.25	Branded ex-factory price [42]
	Brinzolamide + timolol cost (€)	€16.88	Branded ex-factory price [42]
	Dorzolamide + timolol cost (€)	€7.33	Generic ex-factory price [41]
	Travoprost + timolol cost (€)	€18.26	Generic ex-factory price [41]
	Timolol cost (€)	€5.70	Generic ex-factory price [41]
	Tafluprost cost (€)	€25.94	Branded ex-factory price [42]
	Bimatoprost MS (%)	15.6%	[43]
	Bimatoprost + timolol MS (%)	11.5%	
	Brinzolamide + timolol MS (%)	13.9%	
	Dorzolamide + timolol MS (%)	16.4%	
	Travoprost + timolol MS (%)	6.6%	
	Timolol MS (%)	25.4%	
	Tafluprost MS (%)	10.7%	
Disease monitoring	Ophthalmologist consultation cost (€)	€20.66	Code 95.02 [44]
	Gonioscopy cost (€)	€7.75	Code 95.26 [44]
	VF defect test cost (€)	€16.78	Code 95.05 [44]
	Optic disc imaging cost (€)	€90.00	Code: 95.17 [36]
	Incidence ophthalmologist consultation (n/month), mild glaucoma	0.17	[26]
	Incidence ophthalmologist consultation (n/month), moderate glaucoma	0.25	
	Incidence ophthalmologist consultation (n/month), advanced glaucoma	0.33	
	Incidence ophthalmologist consultation (n/month), severe/blind glaucoma	0.25	
	Incidence gonioscopy (n/month), mild glaucoma	0.08	
	Incidence gonioscopy (n/month), moderate glaucoma	0.08	
	Incidence gonioscopy (n/month), advanced glaucoma	0.08	
	Incidence gonioscopy (n/month), severe/blind glaucoma	0.08	
	Incidence VF defect test (n/month), mild glaucoma	0.17	
	Incidence VF defect test (n/month), moderate glaucoma	0.17	
	Incidence VF defect test (n/month), advanced glaucoma	0.25	
	Incidence VF defect test (n/month), severe/blind glaucoma	0.17	
	Incidence optic disc imaging (n/month), mild glaucoma	0.17	
	Incidence optic disc imaging (n/month), moderate glaucoma	0.17	
	Incidence optic disc imaging (n/month), advanced glaucoma	0.33	
	Incidence optic disc imaging (n/month), severe/blind glaucoma	0.17	
Adverse events	Hyperaemia unit cost (€)	€20.66	Code 89.07 [44]
	Stent obstruction unit cost (€)	€1522.00	DH 42 [38]
	Incidence hyperaemia, iStent + cataract group (%)	0.8%	[45]
	Incidence stent obstruction, iStent + cataract group (%)	6.2%	
	Incidence hyperaemia, cataract surgery only group (%)	5.9%	
	Incidence stent obstruction, cataract surgery only group (%)	0.0%	

*DH* day hospital, *MS* market share, *VF* visual field

The cost of cataract surgery was derived from the national tariff of hospital procedures [38].

Some outpatient procedures are available within several regional health systems and carried out in regional health structures with recognized clinical validity but are not available nationally.

For this reason, the costs of certain procedures have been obtained from the regional tariff data.

The cost associated with iStent inject + cataract surgery was calculated based on Glaukos market data and the average costs of procedures in Tuscany, Umbria and Veneto regions [34–36].

The cost of trabeculectomy was calculated as the average cost of interventions (“Trabeculectomy ab externo”) in Friuli Venezia-Giulia, Molise and Veneto regions [35, 39, 40]).

Acquisition costs of glaucoma medical therapy were included in the analysis. Monthly costs of the different medical therapies used in glaucoma were calculated using the ex-factory price (branded or generic) [41, 42]. Then, these costs were multiplied by the respective market shares of these therapies in Italy [43], and finally summed up to determine an average monthly cost of glaucoma medical therapy in Italy.

The costs of glaucoma disease monitoring were included in the model; it was assumed that resource consumption depended on disease severity [26]. The following resources were considered: i) ophthalmologist consultation; ii) gonioscopy; iii) visual field test; iv) optic disc imaging. Finally, the costs for the management of treatment-related adverse events were calculated by multiplying the unit costs in the Italian practice [38, 44], by the respective adverse event rates [16, 45]. Only adverse events with at least 3% difference between the two treatment arms were included. The adverse event costs were one-time costs, applied only at the beginning of the simulation, corresponding with the index intervention.

### Utility inputs

Quality-adjusted life years (QALYs) were estimated as the sum of the life years spent in each health state, weighted by the associated utilities, thus reflecting the average health-related quality of life (HRQOL) of glaucoma patients in each stage of the disease.

Table 3 shows the utilities associated with the health states of the model. These values have been extracted from two studies conducted in 2010 and 2012 in the Netherlands [46, 47], which correlated the loss of vision with health-related quality of life (HRQoL).

**Table 3:** Utilities associated with the health states of the model [46, 47]

Health state	Utility
Mild glaucoma	0.847
Moderate glaucoma	0.781
Advanced glaucoma	0.704
Severe/blind glaucoma	0.594

Disutility for trabeculectomy was estimated from the same study conducted by Van Gestel et al. [47] (Table 4). Disutilities for medication-related adverse events were subtracted to the health state utilities. Such disutility values were multiplied by the probability of experiencing the adverse event, to obtain an average disutility value. Also, since not all patients were receiving medications at each Markov cycle, the disutility value was weighted by the proportion of patients receiving therapy at each Markov cycle. The incidence rates of adverse events in patients receiving prostaglandins, beta-blockers and carbonic anhydrase inhibitors were 8, 8, and 14%, respectively [48]; market shares of these drugs were 35.2, 15.2, and 49.6%, respectively (obtained from the market shares of the single drugs reported in the Table 2). Consequently, the medication weighted incidence of adverse events was 8.9%; the corresponding disutility value was calculated multiplying this percentage by the disutility value for medication-related averse events (Table 4), estimated from the Van Gestel et al. study [47].

**Table 4:** Disutilities included in the model [47]

Description	Disutility	CI (95%)	Source
Trabeculectomy	−0.007	0.005–0.009	[47]
Medication-related AEs	− 0.101	0.076–0.126	

*AEs* adverse events, *CI* confidence interval

### Sensitivity analysis

Deterministic (one-way) and probabilistic sensitivity analyses were carried out to identify the input values with the largest effect on incremental cost-effectiveness ratio (ICER).

For the deterministic sensitivity analysis, the baseline value of each parameter was modified to the upper and lower limits of its 95% confidence interval (95% CI). If the CI was not available, a variation of ±20% from the baseline value was used (Additional file 1).

A probabilistic sensitivity analysis was performed, simultaneously and randomly varying the values of all model parameters (1000 replications). For the probabilistic analysis, the following probability distributions were used: beta for probabilities and utilities; gamma for costs; normal for efficacy data (Additional file 1).

## Results

### Base-case analysis

Results of the base-case analysis are shown in Table 5. iStent *inject*® + concurrent cataract surgery was more effective than cataract surgery alone, in terms of quality-of-life-adjusted survival (11.11 QALYs, compared with 11.02 QALYs respectively). The total lifetime costs were higher in the iStent *inject*® + concurrent cataract surgery, compared with the cataract surgery alone group (€8368.51 vs. €7134.71 respectively). The higher procedural cost and the acquisition costs of iStent *inject*® were the driver of this cost increase. The resulted incremental cost-effectiveness ratio was €13,037.01 per QALY gained.

**Table 5:** Results of cost utility analysis: base-case

• Treatment	iStent ***inject***® + cataract surgery	Cataract surgery
Costs (€)		
• Procedures (€)	€ 3557.00	€ 2317.06
• Medications (€)	€25.59	€51.17
• Progression-related (€)	€4691.45	€4765.25
• Adverse events (€)	€94.46	€1.22
• **Total costs (€)**	**€8368.51**	**€7134.71**
• Efficacy		
• Survival (life years, LYs)	• 14.575	• 14.575
• **Quality of life adjusted survival (QALY)**	• **11.114**	• **11.019**
• Incremental outcomes (iStent *inject*® + cataract surgery vs. cataract surgery alone)		
• Incremental costs (€)	• €1233.80	
• Incremental LYs	• –	
• Incremental QALYs	• 0.095	
• **ICER (€/QALY)**	• **€13,037/QALY**	

*LYs* life years, *QALYs* quality-adjusted life years

### Sensitivity analysis

**Fig. 2:**
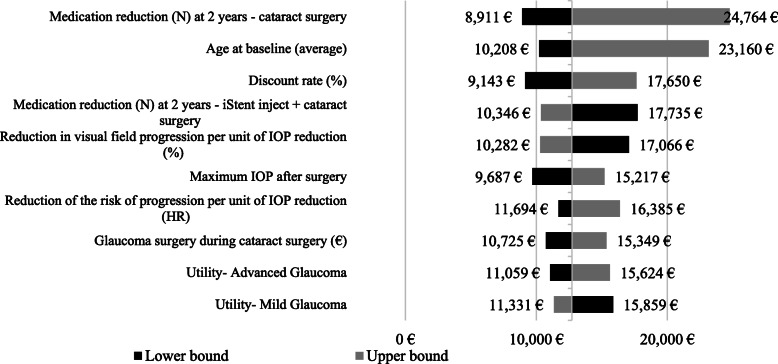
Results of One-way deterministic sensitivity analysis. *ICER = incremental cost-effectiveness ratio; IOP = intraocular pressure; MD = mean deviation; QALY = quality-adjusted life year. The upper/lower bounds of the parameters are shown in the* Additional file 1

Both one-way deterministic and probabilistic sensitivity analyses confirmed the robustness and reliability of base-case results. The results of one-way deterministic analysis are summarized in Fig. 2, that illustrates the 10 parameters / scenarios with the greatest effect on the ICER (base-case ICER: €13,037 / QALY). The ICER variability was modest (minimum ICER: €8911/ QALY earned; maximum ICER: €24,764/ QALY gained).

**Fig. 3:**
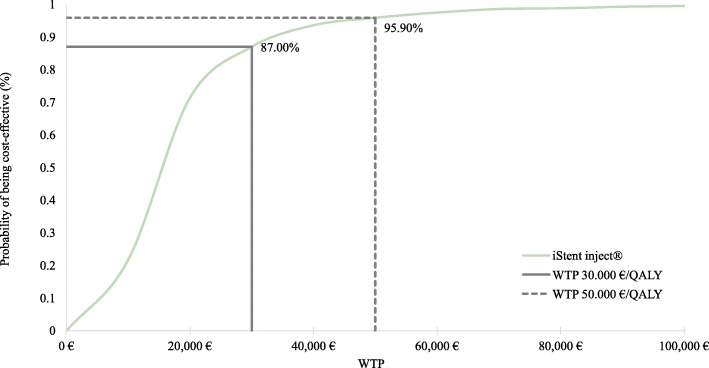
Acceptability Curve for iStent + cataract surgery versus cataract surgery. *QALY = quality-adjusted life year; WTP = willingness to pay*

**Fig. 4:**
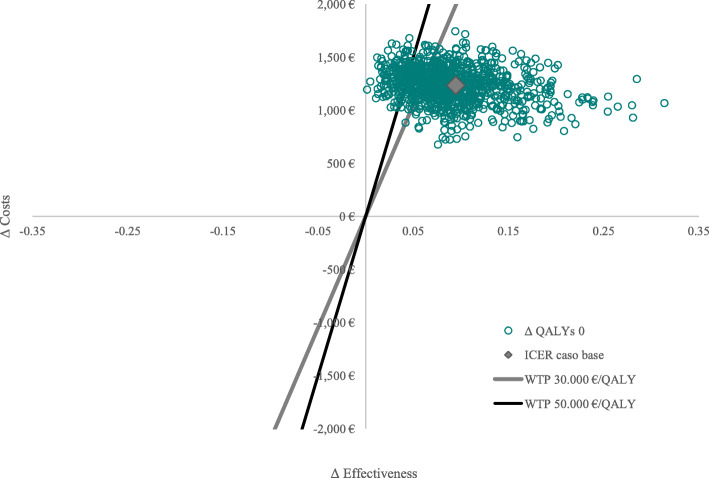
Scatterplot for iStent + cataract surgery versus cataract surgery. *ICER = cost-effectiveness ratio; QALY = quality-adjusted life year; WTP = willingness to pay*

The results of the probabilistic sensitivity analysis are reported in Fig. 3 (acceptability curve of cost-effectiveness –CEAC-) and Fig. 4 (scatterplot). The acceptability curve of cost-effectiveness (CEAC) analysis (Fig. 3) showed that when the willingness to pay (WTP) is equal to €50,000 per QALY gained, iStent *inject*® + cataract surgery would have a 96% probability of being cost-effective, compared to cataract surgery alone.

## Discussion

Glaucoma is not only a major health problem, with a significant impact on patients’ quality of life and social functioning, but also a relevant economic issue for healthcare systems [49]. In Italy, glaucoma management costs are estimate in €0.4 - €0.5 billion yearly of which drugs and specialist consultations were the largest cost components [50]. Importantly, both the clinical and economic burden of the disease increase as glaucomatous damage and vision loss progress [49, 50]. Therefore, any inefficiency in disease diagnosis, delayed treatment or poor IOP control would translate into poor patient prognosis and increased costs for healthcare systems and society.

The recent technological advances in glaucoma management, specifically laser trabeculoplasty and MIGS, offer ophthalmologists new options to manage POAG patients more effectively. Medical therapy has been the mainstay of glaucoma treatment for decades. However, medical treatment can be associated with adverse events, and some patients may be unable to comply adequately with complex dosing regimens [51].

iStent *inject*® has been shown to be effective in the management of patients with mild-moderate glaucoma who also require cataract surgery. The present cost-effectiveness analysis, based on Italian National Health Service (NHS) data, shows that iStent *inject*® is a cost-effective option in patients with POAG, in need of simultaneous cataract intervention.

The cost-effectiveness analysis shows that a moderate QALY gain can be obtained with iStent *inject*® + cataract, with a modest economic investment. Although the use of iStent *inject*® does not seem to produce significant cost-offset (only a slight reduction of progression-related costs has been observed), the total incremental investments are quite low (+€1234) and the overall lifetime costs are quite low as well (€8369 per patient). These costs are much smaller when compared with those of other diseases, where costs of ~€10 K are sustained on an annual basis, rather than on a lifetime basis (e.g. treatment of autoimmune conditions with monoclonal antibodies, target therapies, etc.).

Moreover, the incremental cost-effectiveness ratio of €13,037 per QALY gained is below the Italian informal acceptability threshold, amounting to €25–40 thousands per QALY gain [52].

Along with these economic considerations, there is a clear clinical rationale justifying the place in therapy of iStent *inject*® in glaucoma + cataract [5, 16–19]. First, most of the evidence of iStent *inject*® regards POAG patients in need of cataract surgery. In this setting, it was showed that: i) device implantation is safe (i.e., negligible increase of adverse events, compared to cataract alone); ii) treatment in conjunction with cataract is more effective than cataract alone; iii) treatment effect is durable, thus postponing the need of more invasive glaucoma treatments, such as trabeculectomy.

The fact that iStent *inject*® can be performed during the cataract extraction, prolonging the primary intervention of a few minutes only, poses interesting economic considerations, since the procedural incremental costs associated with MIGS would be minimal in the hospital perspective.

Finally, iStent *inject*® implantation is a valid option to reduce the use of medical therapy. As explained earlier in the discussion, there is a critical, still unmet need of reducing the use of multiple, high-dosage medical treatment. In this context, clinical studies have demonstrated that IOP control can be achieved with iStent *inject*®, either without therapies or with a reduced number of therapies.

Overall, a thorough assessment of the methodological approach used to conduct this analysis is important to check validity of findings. In our view, adoption of conservative assumptions and generalizability of findings are two positive factors supporting the validity of the analysis. Whereas applicable, we opted for conservative assumptions, potentially underestimating cost-effectiveness of iStent *inject*® + cataract surgery. Likely, the most conservative assumption regarded the progressive reduction of IOP control over time (waning effect) for the two study treatments. In the model, a similar waning effect was assumed for iStent *inject*® + cataract surgery and cataract surgery alone. While the progressive loss of therapeutic effect is clearly documented in literature for patients receiving cataract surgery [14], this might be delayed with iStent *inject*® + cataract surgery; however, no difference was modelled, given the uncertainty on this potential benefit. As regards generalizability of findings, instead, it should be remarked that the analysis is based on the randomized clinical trial (RCT) conducted by Samuelson et al. 2019 [16], enrolling patients with 24.8 mmHg + 3.3. This proves that iStent *inject*® + cataract was more effective than cataract alone in a large variety of patients, even in those with quite high IOP at baseline.

Together with the above-mentioned analysis strength, a few limitations exist in this analysis.

First, someone could argue that a comparison between iStent *inject*® + cataract, vs. cataract followed by laser trabeculoplasty, would be more appropriate for decision-making processes. However, the lack of direct evidence comparing TBS vs. trabeculoplasty would make this comparison hard to conduct, to date. We expect this comparison would be critical for decision making in the future when appropriate evidence will be collected. Today, with the currently available information, we can only suppose that TBS might be either dominant or cost-effective than laser trabeculoplasty, because of the more robust long-term evidence, which is the main weakness of the latter [53].

Second, it would be interesting to conduct a cost-utility analysis of iStent *inject*®, looking at the social costs, rather than third-payer expenses only. Therefore, future assessments should evaluate this perspective as well.

Third, in this analysis, several assumptions were formulated through experts’ opinion, due to the lack of published literature or limited information on the Italian practice. We acknowledge this as a potential limitation of the analysis, that was partially managed conducting sensitivity analysis. However, an update of this analysis is recommended as soon as data relating to the Italian context will be available.

The third, and probably most important point to assess would regard the economic sustainability of iStent *inject*® for hospitals. In our base-case analysis, the cost of the iStent inject® implantation was used in conjunction with cataract surgery: €2294, (i.e. average of the tariffs of the procedure “Other interventions for glaucoma” [34–36] + iStent *inject*® acquisition costs). With these cost assumptions, iStent *inject*® + cataract was cost-effective vs. cataract alone. However, Italian hospitals are not receiving this remuneration for this combined intervention to date. Hospitals could obtain a remuneration of up to €1522, which is the day-hospital intervention for glaucoma (DH 42 “Interventions on intraocular structures except retina, iris, and crystalline”) [38]. In other words, with the current remuneration levels, hospitals would not be able to afford overall costs (iStent Inject®, cataract intraocular lenses, additional procedural charges -room, staffing, other equipment-, etc.). However, this intervention would be cost-effective for the Italian NHS. Our aim, with this paper, is then to make aware budget holders about this economic “paradox” and to evaluate solutions aimed at solving this issue. The future analyses on iStent Inject® should then assess both the NHS and hospital perspectives and verify whether results would be advantageous for both parties.

## Conclusions

The results of the cost-utility analysis confirm that iStent *inject*® + cataract surgery is a cost-effective option for the treatment of patients affected by mild-to-moderate POAG, compared with cataract surgery alone, based on Italian NHS data.

## Supplementary Information

**Additional file 1.** Parameters included in the analysis.

## Data Availability

Not applicable.

## Abbreviations

AEs: Adverse events
ALT/SLT: Laser trabeculoplasty
CEAC: Acceptability curve of cost-effectiveness
CI: Confidence interval
dB: decibel
DH: Day hospital
EMGT: Early Manifest Glaucoma Trial
HRQOL: Health-related quality of life
IOP: Intraocular pressure
ICER: Incremental cost-effectiveness ratio
ISTAT: Italian Institute of Statistics
MD: Mean deviation
MIGS: Micro-invasive glaucoma surgery
MS: Market share
NHS: National Health Service
NICE: National Institute of Health Care and Excellence
OAG: Open-angle glaucoma
POAG: Primary open-angle glaucoma
QALY: Quality-adjusted life years
RCT: Randomized clinical trial
TBS: Trabecular bypass stent
US: United States
VF: Visual field
WTP: Willingness to pay
