# Supplementary material for: Cost-utility analysis of trabecular micro-bypass stents (TBS) in patients with mild-to-moderate open-angle Glaucoma in Italy
Source: BMC Health Serv Res. 2021 Aug 17;21:824. doi: 10.1186/s12913-021-06862-x (PMC8369731; doi:10.1186/s12913-021-06862-x)
Supplement: Supplementary file 1 — Additional file 1. Parameters included in the analysis. [file 12913_2021_6862_MOESM1_ESM.docx]

Additional file 1 Parameters included in the analysis.

| **Parameter** | **Point estimate** | **Range of variation** | | **Distribution** |
| --- | --- | --- | --- | --- |
|  |  | **Lower bound** | **Upper bound** |  |
| Discount rate (%) | 3.5% | 0% | 7% | Not included in PSA |
| Baseline age (mean) | 64.7 | 51.76 | 77.64 | Lognormal |
| Patients entering with mild glaucoma (%) | 0.5 | 0.4 | 0.6 | Beta |
| Reduction in decline in visual field per unit reduction in IOP (mmHg) | 0.095 | 0.076 | 0.114 | Beta |
| Decrease in visual field after one year of no treatment (dB) | -0.0508 | -0.063 | -0.039 | Normal |
| Reduction of the risk of progression per unit of IOP reduction (HR) | 0.885 | 0.708 | 0.973 | Lognormal |
| IOP reduction at 1 year- iStent inject + cataract surgery (mmHg) | 8.4 | 8.047 | 8.753 | Normal |
| IOP reduction at 2 years - iStent inject + cataract surgery (mmHg) | 8.3 | 7.927 | 8.673 | Normal |
| Medication reduction per year- iStent inject + cataract surgery (N) | 1.3 | 1.197 | 1.403 | Normal |
| Medication reduction at 2 years - iStent inject + cataract surgery (N) | 1.2 | 1.080 | 1.320 | Normal |
| IOP reduction at one year - cataract surgery (mmHg) | 8.3 | 7.509 | 9.091 | Normal |
| IOP reduction at 2 years - cataract surgery (mmHg) | 8.1 | 7.283 | 8.917 | Normal |
| Medication reduction per year- cataract surgery (N) | 0.9 | 0.714 | 1.086 | Normal |
| Medication reduction at 2 years - cataract surgery (N) | 0.7 | 0.445 | 0.955 | Normal |
| Maximum IOP after surgery (mmHg) | 17 | 14 | 21 | Not included in PSA |
| Extrapolation - reduction in efficiency (N) | 0.067 | 0.05 | 0.1 | Not included in PSA |
| IOP reduction – Trabeculectomy (mmHg) | 6.48 | 6.396 | 6.564 | Normal |
| Hyperemia- iStent + cataract surgery (N) | 0.008 | 0.0064 | 0.0096 | Beta |
| Stent obstruction- iStent + cataract surgery (N) | 0.062 | 0.0496 | 0.0744 | Beta |
| Hyperemia- cataract surgery (N) | 0.059 | 0.0472 | 0.0708 | Beta |
| Stent obstruction- cataract surgery (N) | 0 | 0 | 0 | Beta |
| No. visits to the ophthalmologist per year- severe Glaucoma / blindness (N) | 3 | 2.4 | 3.6 | Lognormal |
| No. gonioscopies per year- severe Glaucoma / blindness (N) | 1 | 0.8 | 1.2 | Lognormal |
| No. of visual acuity tests per year- severe Glaucoma / blindness (N) | 2 | 1.6 | 2.4 | Lognormal |
| No. of optic disc images per year- severe Glaucoma / blindness (N) | 2 | 1.6 | 2.4 | Lognormal |
| Cost of medication per month (€) | 10.69 | 8.55 | 12.83 | Gamma |
| Cataract surgery cost (€) | 994.0 | 795.2 | 1,192.8 | Gamma |
| Glaucoma surgery during cataract surgery + iStent inject (€) | 2,294.24 | 1,835.4 | 2,753.1 | Gamma |
| Cost of trabeculectomy (€) | 1,969.05 | 1,575.24 | 2,362.86 | Gamma |
| Cost of the visit to the ophthalmologist (€ / unit) | 20.66 | 16.53 | 24.79 | Gamma |
| Gonioscopy cost (€ / unit) | 7.75 | 6.2 | 9.3 | Gamma |
| Cost of the visual acuity test (€ / unit) | 16.78 | 13.42 | 20.14 | Gamma |
| Optical disc imaging cost (€ / unit) | 90 | 72 | 108 | Gamma |
| Cost of hyperemia (€ / unit) | 20.66 | 16.53 | 24.79 | Gamma |
| Cost of stent obstruction (€ / unit) | 1,522 | 1,217.6 | 1,826.4 | Gamma |
| Utility - Mild glaucoma (N) | 0.847 | 0.801 | 0.886 | Beta |
| Utility - Moderate glaucoma (N) | 0.781 | 0.729 | 0.827 | Beta |
| Utility - Advanced glaucoma (N) | 0.704 | 0.648 | 0.755 | Beta |
| Utility - Severe glaucoma / blindness (N) | 0.594 | 0.536 | 0.650 | Beta |
| Disutility- trabeculectomy (N) | 0.007 | 0.001 | 0.025 | Beta |
| Disutility - use of medication (N) | 0.101 | 0.069 | 0.141 | Beta |

HR: hazard ratio; IOP: intraocular pressure.
